# Kinematic Analysis of Postural Stability During Ballet Turns (*pirouettes*) in Experienced and Novice Dancers

**DOI:** 10.3389/fbioe.2019.00290

**Published:** 2019-10-25

**Authors:** Chai-Wei Lin, Fong-Chin Su, Cheng-Feng Lin

**Affiliations:** ^1^Department of Biomedical Engineering, College of Engineering, National Cheng Kung University, Tainan, Taiwan; ^2^Department of Physical Therapy, Shu Zen Junior College of Medicine and Management Kaohsiung, Kaohsiung, Taiwan; ^3^Musculoskeletal Research Center, National Cheng Kung University, Tainan, Taiwan; ^4^Department of Physical Therapy, College of Medicine, National Cheng Kung University, Tainan, Taiwan; ^5^Institute of Allied Health Sciences, College of Medicine, National Cheng Kung University, Tainan, Taiwan

**Keywords:** center of mass, center of pressure, balance, inclination angle, ballet, posture

## Abstract

Turning is an important but difficult movement, often performed in ballet choreography. Understanding the postural sway during ballet turns is beneficial to both dancers and dance teachers alike. Accordingly, this study evaluated the postural sway angle during ballet turns in female novice and experienced ballet dancers by means of the inclination angle, determined from the center of mass (COM) and center of pressure (COP). Thirteen experienced dancers and 13 novice dancers performed ballet turns (*pirouettes*). The COM-COP inclination angle was measured during the preparatory, double-leg support, and single-leg support phases of the turn. The novice dancers exhibited significantly greater ranges of the COM-COP inclination angle in the anterior-posterior (AP) and medial-lateral (ML) directions during the preparatory (AP direction, *p* < 0.001; ML direction *p* = 0.035), double-leg support (AP direction *p* < 0.038; ML direction *p* = 0.011), and ending phases (AP direction *p* < 0.001; ML direction *p* = 0.024). Moreover, during the preparatory phase, the novice dancers failed to adjust their posture in a timely manner, and therefore showed overshooting errors. Finally, during the ending phase, the novice dancers showed a greater standard deviation of the COM-COP inclination angles and performed continual postural adjustments, leading to a less smooth movement than the experienced dancers. In conclusion, the novice dancers were suggested to focus on the COM-COP adjustment during both preparatory and ending phases.

## Introduction

Ballet turns, known as *pirouettes*, require whole body rotation on the support of a single leg. *Pirouettes* are a complex movement and require extensive training and accumulated experience. Consequently, the quality of their performance is a function of the dancer's skill level. Although several studies have investigated various characteristics of ballet turns, such as coordination between the upper and lower trunk (Laws, 1978; Owen and Whiting, 1989; Sugano and Laws, 2002; Golomer et al., 2009), the whole body postural sway at different phases of the *pirouette* movement is still unclear.

It is essential for ballet dancers to maintain dynamic stability of the whole body with an appropriate posture in ballet choreography. A previous study found that the dancers who have the ability to complete multiple-turn *pirouettes* allow their bodies to make adjustments throughout the turn instead of maintaining a rigid trunk (Lott and Laws, 2012). When performing a *pirouette*, dancers raise the heel of the supporting leg to the single-leg *demi-pointe* position (i.e., standing on the ball of the foot) to reduce friction during turning. During the actual turning phase (i.e., single-leg support), executing the turn requires a proper control of the center of mass (COM) over a small base of support. Specifically, a precise vertical alignment of the COM with the center of pressure (COP) is required to prevent unexpected torque and subsequent loss of balance. Regulation of proper torque and the coordination of each body segment is important for a high-skilled turn (Imura and Yeadon, 2010). Achieving this postural control requires the activation of the muscles around the torso (Winter, 2005) and the muscles affect ground reaction force. However, postural stability must be disrupted as the dancer transits from the initial double-leg support state to the single-leg support state. Consequently, the ability of the dancer to rebuild equilibrium during this transition phase is of critical importance in determining the success of the movement.

The COP displacement is an important parameter in assessing postural stability in many static standing tasks (Lyon and Day, 1997; Hiller et al., 2004; McKeon and Hertel, 2008; Catena et al., 2009). Generally speaking, a larger COP displacement indicates a more unstable posture. However, in dynamic tasks, a larger COP displacement does not necessarily indicate a greater instability. Previous studies find that dancers regulate their reaction force to minimize the COM horizontal velocity to make vertical alignment within the base of support as the number of turns increase (Zaferiou et al., 2016a,b). Thus, it is important to consider not only the COP parameters, but also the COM parameters when exploring postural control during dynamic movements. Additionally, many studies have shown that maintaining postural stability becomes more difficult as the distance between the COP and COM in the horizontal plane increases (Hahn and Chou, 2004; Hsue et al., 2009a,b). The relative arrangement of the COM and COP provides an effective means of gauging the risk of falls in the elderly and children with balance dysfunction (Corriveau et al., 2001; Hahn and Chou, 2004; Hsue et al., 2009a,b). A previous study investigated the angle between the vertical vector and the vector of COP to COM in the turning phase of the single and double *pirouette* and the average angles of COM-COP inclination angle was reported 4 degrees in both single and double turns in their study (Zaferiou et al., 2016b). Furthermore, the angle of the line connecting the COM and the COP with respect to the fixed global vertical axis, the inclination angle, is strongly related to the postural stability condition in dynamic movements (Chen and Chou, 2010). This may further suggest that the greater inclination angle indicates greater risk of falls and less stability in *pirouette*.

Regarding the differences between different skill levels of dancers in ballet turns, the novice dancers spend more preparation time to initiate a turning movement and lack of head spotting technique during *pirouette* (Laws, 1978; Lin et al., 2014). Blanco et al. (2019) presents that a higher correlation between ballet jump and regular jump was found as skill level of dancers increases. Studies also present that dancers had better performance during one-leg stance or during walking than the novice dancers or untrained ones, and thus suggested that longer years of ballet training may be a factor leading to experienced dancers having a superior ability in postural control (Lung et al., 2008; Kilroy et al., 2016). However, the literature lacks information regarding differences in postural stability of dancers with different skill levels during ballet turns. Such information of postural stability is of great interest to dance educators in understanding how best to improve postural stability in novice dancers and to design their training programs accordingly. Therefore, the present study compares the postural stability of experienced and novice dancers at different phases of the ballet turn, using the COM-COP inclination angle and COM-ankle inclination angle as performance indicators. In conducting the investigation, it is hypothesized that experienced dancers exhibit smaller ranges of both inclination angles than novice dancers during all phases of the *pirouette* movement.

## Methods

### Participants

Thirteen experienced female dancers (age: 17.8 ± 3.4 years, height: 159.3 ± 4.2 cm, weight: 51.54 ± 4.66 kg) and 13 novice female dancers (age: 12.0 ± 1.9 years, height: 151.9 ± 11.5, weight: 43.81 ± 9.68 kg) participated in the study. The inclusion criteria for the experienced group were specified as follows: (1) a minimum ballet training history of 6 years (8.7 ± 3.3 years); (2) a minimum of 3 h routine ballet training per week; and (3) the ability to perform double-revolution turns (or more) on single-leg support. The inclusion criteria for the novice group were specified as: (1) a ballet training history of 2–5 years (3.2 ± 1.7 years); (2) a minimum of 1.5 h routine ballet training per week; and (3) the ability to perform complete single-revolution turns on single-leg support. Dancers with vestibular or balance problems, or lower back and lower extremity injuries, were excluded from both groups. Before participating in the study, each participant read and signed an informed consent form approved by the Institutional Review Board of the University Hospital.

### Instrumentation

A real-time motion capture system (200 Hz) with eight Eagle CCD cameras (Motion Analysis Corporation, Santa Rosa, CA, USA) was used to collect the three-dimensional (3D) trajectories of a modified Helen Hayes marker set consisting of 43 reflective markers. The markers were placed on the forehead, top head, rear head, sternal notch, xiphoid process, 7th cervical spinal process (C7), sacrum, midpoints of each arm and forearm, lateral epicondyles of both humeri, radial styloid process, third metacarpal head, both sides of the anterior superior iliac spine (ASIS), midpoints of each thigh and shank, greater trochanters, lateral knee joint lines, lateral malleoli, midpoints between 1st and 5th metatarsal heads, and heel posteriors, respectively. The markers were attached either to the participant directly or to the leotard or soft shoes. Two static standing trials with an additional eight markers were conducted before the dynamic trials in order to calculate the joint centers. The additional markers were placed bilaterally on the medial humeral epicondyles, ulnar styloid processes, medial knee joint lines and medial malleoli, and were removed during the dynamic trials. The markers were placed by the same individual for all the trials and participants.

The ground reaction force (GRF) during the *pirouette* turn was measured using two 60 × 40 cm force plates (9281B, Kistler Instrument Corp., Winterthur, Switzerland) synchronized with the motion capture system and sampled at a frequency of 1,000 Hz. To ensure the accuracy of the GRF measurements, the force plates were physically isolated from their surroundings and the performance area was cleaned and expanded by wooden plates. Moreover, the performance area was covered with vinyl to simulate the floor condition in a typical ballet classroom.

### Procedures

**Figure 1:**
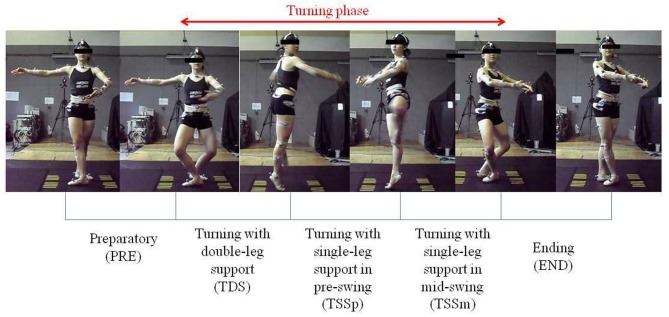
Five phases in *pirouette en dehors*. (Written informed consent for the publication of this image was obtained).

Each participant performed five single-revolution *pirouette en dehors* using the dominant leg as support (Figure 1). Note that the dominant leg was defined as the leg used by the participants to kick an object, and was found to be the right leg in every case. The reason for choosing the dominant leg as the support leg was because the differences between novice and experienced dancers during ballet turns were greater in the dominant leg support. That means ballet turning with the dominant leg is more difficult for dancers. The marker trajectories and GRF data were recorded continuously throughout the turn. At the beginning of each trial, the participants were requested to adopt the ballet *fourth position* with the gesture leg behind the supporting leg. In response to an auditory cue, the participants flexed their knees as preparatory movement and raised the gesture leg to the ballet *retire position* (i.e., the foot of the gesture leg placed near the medial knee joint line of the supporting leg). The participants then performed a single-revolution *pirouette en dehors*. Upon completion of the *pirouette*, the participants landed in the ballet fifth position (i.e., the gesture leg placed closely behind the supporting leg) and returned to the upright position.

### Data Analysis

The *pirouette en dehors* movement was subdivided into three phases, namely preparatory, turning and ending (Figure 1 and Table 1). The turning phase was further divided into three sub-phases, i.e., turning with double-leg support, turning with single-leg support in pre-swing, and turning with single-leg support in mid-swing. The duration of each phase was determined manually from the images captured by the CCD cameras. In analyzing the movement, single-leg support in the pre-swing phase was assumed to begin when the gesture leg came off the force plate, and continued until the *retire position* was reached. The *retire position* was determined by the least distance between the toe marker on the gesture leg and the virtual marker representing the medial knee joint line on the supporting leg.

**Table 1:** Determination of each phase.

**Phase**	**Start**	**Detected by**	**End**	**Detected by**
Preparation	Supporting leg knee starts to flex	Knee flexion angle of supporting leg changes	Upper extremities apart	Initiation of the side of upper extremity toward turning direction in the horizontal plane
Double support	Upper extremities go apart	Initiation of the side of upper extremity toward the turning direction in horizontal plane	Gesture leg push off	Gesture leg off the force plate
Pre-swing	Gesture leg pushes off	Gesture leg off the force plate	Gesture leg on *retire* position	Smallest distance between toe marker on gesture leg and virtual marker on medial knee joint line of supporting leg
Mid-swing	Gesture leg is on *retire* position	Smallest distance between toe marker on gesture leg and virtual marker on medial knee joint line of supporting leg	Gesture leg down	Heel marker on gesture leg in vertical direction back to starting position
Ending	Gesture leg downs	Heel marker on gesture leg in vertical direction back to starting position	Bilateral knee in extension	Supporting leg knee extension angle backs to starting angle

The COP during the *pirouette en dehors* movement was calculated as:

(1) $$\begin{array}{r} Resultant\;COP=\frac{F_{z1}}{F_{z1}+F_{z2}}\times COP_{1}+\;\frac{F_{z2}}{F_{z1}+F_{z2}}\times COP_{2} \end{array}$$

where, *F*_*z*_ is the vertical GRF and the lower-case suffixes denote the 1st and 2nd force plates, respectively (Winter, 2005). Note that the COP positions are all expressed with respect to the global coordinate system.

**Figure 2:**
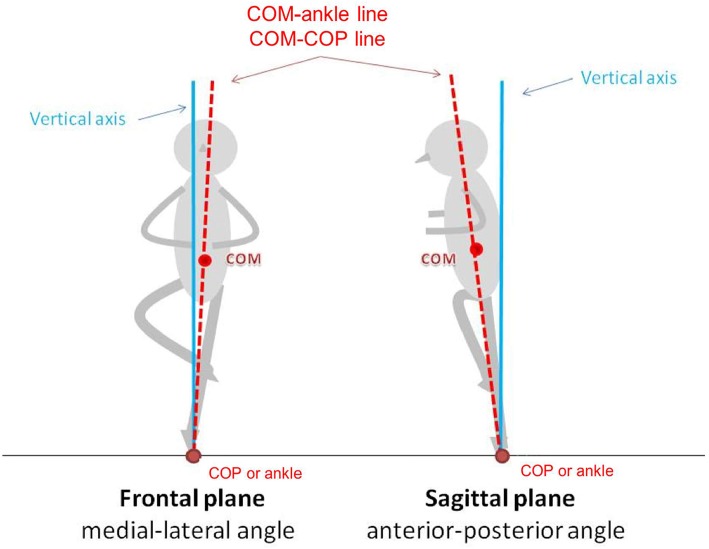
COM–COP and COM-ankle inclination angles in the frontal and sagittal planes.

The whole body COM was calculated using a 13-segment model consisting of the head-neck, upper arms, trunk, forearm-hands, pelvis, thighs, shanks and feet. The estimated COM of each segment was determined from the 3D locations of the respective markers and the anthropometry data provided in Dempster's model (Winter, 2005). The calculated whole body COM position was then transformed to a local coordinate (pelvic coordinate) frame constructed in accordance with the markers on the sacrum and bilateral ASISs. The COM-COP inclination angles in the anterior-posterior and medial-lateral directions, relative to the pelvic orientation, were calculated from the relative positions of the COM and COP, and the vertical height of the COM. The medial-lateral inclination angle (inclination_ML_, Equation 2) was used to evaluate the sway angle in the frontal plane, while the anterior-posterior inclination angle (inclination_AP_, Equation 3) was used to evaluate the sway angle in the sagittal plane [Figure 2; (Chen and Chou, 2010)]. Note that the medial direction was measured in the direction toward the side of the supporting leg, while the lateral direction was measured in the direction toward the side of the gesture leg.

(2) $$\begin{array}{l} inclination_{ML}=\\ \begin{array}{c} \tan^{-1}\frac{Relative\;position\;of\;COM\;and\;COP\;in\;ML\;direction}{Height\;of\;COM\;} \end{array} \end{array}$$

(3) $$\begin{array}{l} inclination_{AP}=\\ \begin{array}{c} tan^{-1}\frac{Relative\;position\;ofCOM\;and\;COP\;in\;AP\;direction}{Height\;of\;COM\;} \end{array} \end{array}$$

Some participants were observed to use a leaping strategy in the single-leg support phase of the *pirouette* movement. Consequently, both feet left contact with the force plates, and hence, the COM-COP relationship could not be applied to evaluate the postural sway. Hence, for this particular phase of the movement, the COP position was substituted by the midpoint position between the ankle joint center and the metatarsal marker. In other words, for all participants, the COM-COP inclination was measured in the double-leg support phases of the *pirouette en dehors* movement (i.e., the preparatory, double-leg support, and ending phases), while the COM-ankle inclination angle was measured in the single-leg support phases (i.e., the pre-swing and mid-swing phases). For all of the phases, the COM and COP angle data were time normalized to 100% with 101 time points.

Each participant performed five *pirouettes* and was awarded a score for each trial by each participant and a judge with extensive ballet choreography experience. The scores were assigned in the range of 1–5; with a value of 1 indicating a poor performance and 5 an excellent performance. For each participant, the trials awarded the three highest scores were taken for subsequent analysis purposes. The ranges of the medial-lateral and anterior-posterior direction of COM-COP angles in the preparatory, turning with double-leg support and ending phases, and the ranges of COM-ankle inclination angles in the single-leg support phases were calculated. Moreover, the maximum inclination angle was detected in the three turning phases (i.e., double-leg support, pre-swing and mid-swing). In addition, the COM-ankle inclination angles at the *retire position* were also measured. Finally, for each participant, the standard deviations of the COM-COP inclination angles were calculated during the ending phase. All of the variables were analyzed using standard SPSS 17.0 statistical software (SPSS for Windows, Chicago, IL, USA). The Cohen's *d* effect size was calculated by dividing the mean difference by their pooled standard deviation. Significant differences between the two groups were detected by performing independent *t*-tests with a significance level of α < 0.05.

## Results

The novice group exhibited significantly greater ranges of COM-COP inclination_AP_ and inclination_ML_ during the preparatory, double-leg support and ending phases. No significant difference was observed in the range of COM-ankle inclination_AP_ for the two groups in the pre-swing phase. However, in the mid-swing phase, the range of COM-ankle inclination_AP_ for the novice group was significantly larger than that of the experienced dancers. Finally, no statistical difference was noted between the two groups in the range of COM-ankle inclination_ML_ during the pre-swing and mid-swing phases (Table 2).

**Table 2:** Range (SD) of COM-COP (PRE, TDS) and COM-ankle (TSSp, TSSm) inclination angles (degrees).

	**Experienced**	**Novice**	***p*-value**	**Effect size**
**Anterior-posterior Direction**				
PRE	2.77 (2.58)	5.62 (3.82)	< 0.001^*^	0.87
TDS	11.25 (2.80)	12.82 (3.50)	0.038^*^	0.50
TSSp	9.21 (2.17)	9.00 (2.18)	0.682	0.10
TSSm	4.97 (2.17)	7.76 (3.79)	< 0.001^*^	0.90
END	5.16 (2.20)	9.73 (5.00)	< 0.001^*^	1.18
**Medial-lateral Direction**				
PRE	0.03 (0.03)	0.08 (0.16)	0.035^*^	0.43
TDS	0.05 (0.01)	0.11 (0.13)	0.011^*^	0.65
TSSp	0.02 (0.01)	0.02 (0.03)	0.756	< 0.01
TSSm	0.05 (0.09)	0.06 (0.09)	0.547	0.11
END	0.05 (0.02)	0.17 (0.28)	0.024^*^	0.60

* *Significant difference between groups (p < 0.05); PRE, preparatory phase; TDS, turning with double leg support; TSSp, Turning with single leg support in pre-swing; TSSm, Turning with single leg support in mid-swing; END, ending phase*.

The novice dancers showed significantly greater maximum anterior, medial and lateral inclination angles than the experienced dancers during the turning phase (i.e., double-leg support, pre-swing, and mid-swing). However, the maximum posterior angle was similar for the two groups (Table 3). In the *retire position*; the novice dancers exhibited a greater range of COM-ankle inclination angle in the medial direction but a smaller range of COM-ankle inclination angle in the posterior direction than the experienced dancers (Table 4).

**Table 3:** Maximum inclination angles during turning phase (degrees).

	**Experienced**	**Novice**	***p*-value**	**Effect size**
Anterior	5.38 (1.03)	7.22 (2.36)	< 0.001^*^	1.01
Posterior	8.18 (2.06)	8.92 (2.06)	0.132	0.36
Support leg side	0.01 (0.00)	0.02 (0.01)	0.008^*^	0.36
Gesture leg side	0.05 (0.02)	0.09 (0.08)	0.005^*^	0.69

* *Significant difference between groups (p < 0.05)*.

**Table 4:** COM-ankle inclination angles at *retire* position (degrees).

	**Experienced**	**Novice**	***p*-value**	**Effect size**
Anterior (+) –posterior (–)	−3.72 (1.57)	−1.77 (2.69)	0.001^*^	0.89
Medial (+) –lateral (–)	0.01 (0.01)	0.02 (0.01)	0.001^*^	1.00

* *Significant difference between groups (p < 0.05)*.

During the ending phase, the novice dancers showed a greater standard deviation of both the COM-COP inclination_AP_ angle (experienced: 1.36 ± 0.54°, novice: 2.63 ± 2.15°, *p* = 0.002) and the COM-COP inclination_ML_ angle (experienced: 0.01 ± 0.00°, novice: 0.02 ± 0.02°, *p* = 0.002).

## Discussion

The present findings show that the novice dancers performed *pirouette en dehors* with greater inclination angles than the experienced dancers. In particular, the COM-COP inclination_AP_ angles of the novice group were significantly greater than those of the experienced group in the preparatory, double-leg support, mid-swing and ending phases; while the COM-COP inclination_ML_ angles of the novice group were significantly greater than those of the experienced group in the preparatory, double-leg support and ending phases.

### Preparatory Phase

**Figure 3:**
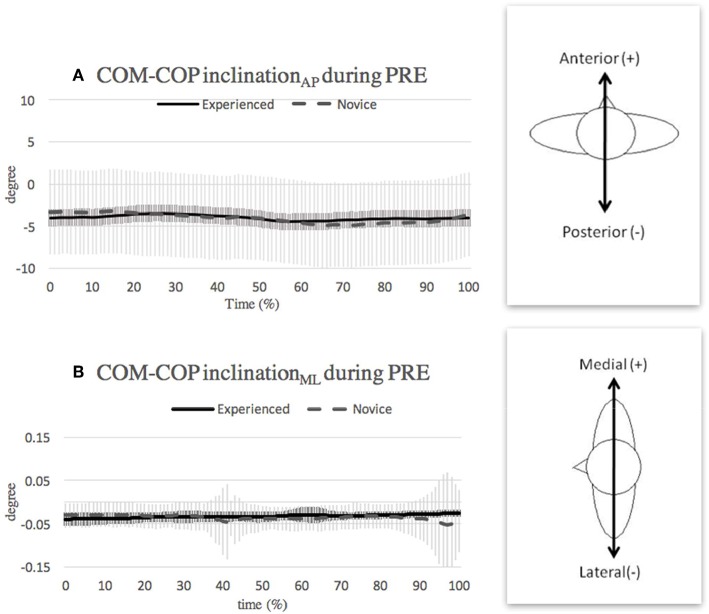
Mean and SD of COM-COP inclination angle in **(A)** anterior-posterior direction and **(B)** medial-lateral direction during preparatory (PRE). Note that the curves show the average value obtained over the three highest-scoring trials of each individual in the respective group.

During the preparatory phase, both groups of dancers lowered their COM by flexing the hips and knees and dorsiflexing the ankles. The dancers started with a center COM, then shifted the weight forward in preparation for single leg stance on the front leg, causing a slight anterior shift in the COP and also in the COM. However, the COP responded faster than the COM, so that a greater anterior shift of the COP than the COM was found at the transition of initiation. As a result, for the 20–55% period of the phase, the COM-COP inclination angle, which already had a slight posterior inclination (COM was behind the COP), increased slightly in the posterior direction (Figure 3). In response, the experienced dancers adjusted their COM-COP inclination angle toward the anterior direction, in order to prepare for the initiation of the turn (55–65% of the phase duration) by shifting their weight toward supporting leg (front leg). However, the novice dancers were less efficient in adjusting their postural sway, and exhibited overshooting errors (at 65% of the phase duration). As a result, the novice dancers exhibited a greater range of COM-COP inclination_AP_ angle than the experienced dancers. Note that this finding is consistent with that of a previous study, which showed that novice golfers have less accuracy in putting than experts due to a poorer recalibration ability (van Lier et al., 2011). The recalibration refers to the ability of perceiving external changes and adjusting accordingly, and this ability can be achieved through enhanced neuromuscular control training (Kiefer et al., 2011). Experienced dancers often have better perceptual sensitivity, and thus better ability to perceive external changes and response to the changes. Ballet practice with continuous inputs of recalibration (i.e., seeing themselves in the mirror) and perceived changes (i.e., dance educators' cues) influences postural control in dancers.

### Turning With Double-Leg Support Phase

**Figure 4:**
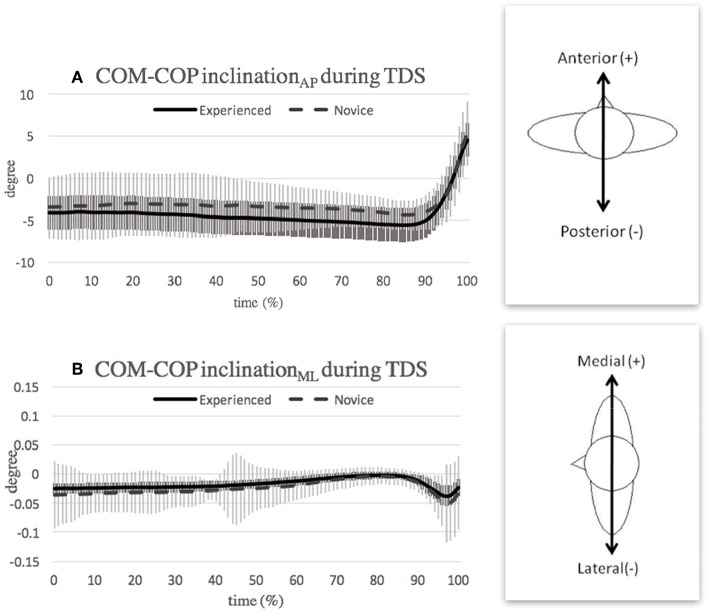
Mean and SD of COM-COP inclination angle in **(A)** anterior-posterior direction and **(B)** medial-lateral direction during turning with double-leg support (TDS) phase. Note that the curves show the average value obtained over the three highest-scoring trials of each individual in the respective group.

For the first 80% of the double-leg support phase, the COM-COP inclination_AP_ angle (Figure 4A) and inclination_ML_ angle (Figure 4B) remained relatively stable in both groups. This finding suggests that most of the movement in the double-leg support phase of *pirouette en dehors* is contributed mainly from motion of the upper extremities and axial rotation of the upper trunk. In a previous study, Kim et al. (2014) showed that dancers apply a twisting motion of the trunk relative to the pelvis, in order to generate additional angular momentum when initiating the turning phase of *pirouette en dehors* (Kim et al., 2014). Thus, both studies confirm the importance of axial trunk motion in performing ballet turns.

At the end of the double-leg support phase (90–100% of the phase), the COM-COP inclination angle moved toward the anterior direction in both groups. This tendency can be attributed to two main factors. First, a ballet *fourth foot position* was requested at the beginning of the task [i.e., the gesture leg (back leg) placed behind the support leg (front leg) with a foot distance apart and the heel of the front leg should be in line with the toes of rear leg]. However, following rotation of the trunk through approximately half a turn, the supporting leg (back leg) was positioned behind the gesture leg (front leg). Note that the front and back legs were defined based on the reference of dancer's trunk. To prepare for gesture leg takeoff, the dancers gradually reduced the weight bearing on the gesture leg; causing the COP to move in a posterior direction toward the supporting leg, and hence the COM-COP inclination angle to increase in the anterior direction. Second, the COM position also contributes to the increased anterior COM-COP inclination angle since the COM is still located anteriorly at the end of the support phase due to its slower response than the COP (Winter, 2005).

The greater range of COM-COP inclination_AP_ angle, during the double-leg support phase in the novice group, may result from the use of a longer preparatory distance (anterior-posterior distance between feet) in performing the *pirouette en dehors* (novice: 190.0 ± 68.0 mm, experienced: 164.6 ± 46.1 mm). The greater distance increases the difficulty to maintain balance during turning because the longer distance between COM and COP needs to be overcome. The results presented in Figure 4B show that the experienced dancers responded more quickly to the subtle change in the lateral direction than the novice dancers. In other words, it appears that the experienced dancers have more precise COM trajectory during double stance that less correction was needed during the turning phase than novice dancers.

### Turning With Single-Leg Support in Pre-swing Phase

**Figure 5:**
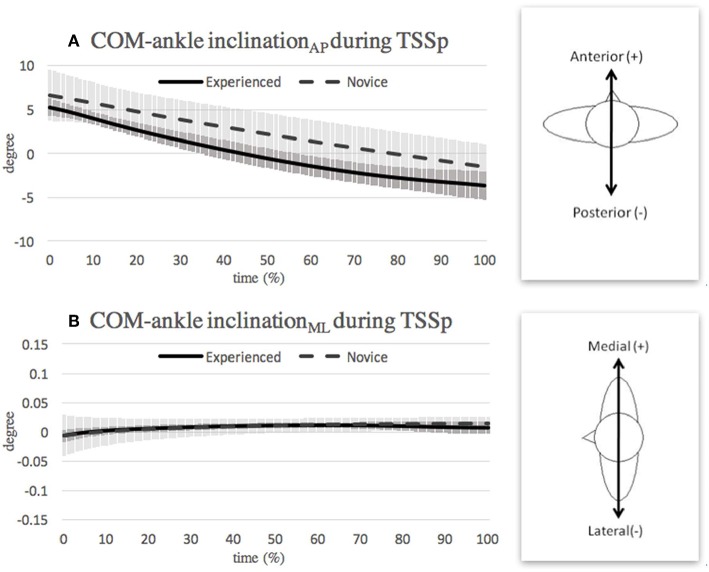
Mean and SD of COM-ankle inclination angle in **(A)** anterior-posterior direction and **(B)** medial-lateral direction during turning with single-leg support in pre-swing (TSSp) phase. Note that the curves show the average value obtained over the three highest-scoring trials of each individual in the respective group.

Although no significant difference was observed between the two groups in the range of inclination_AP_ during the pre-swing phase, the difference in the absolute degrees of the inclination_AP_ angle (Figure 5A) may denote the use of different adjustment strategies. For example, while both groups centralize their COM during the transition from double-leg support to single-leg support, some differences between the two groups may result from an adjustment of the upper extremities and trunk. These differences, and the impact of the upper extremities, require further investigation in a future study. Figure 5B shows that the novice group applied a greater medial COM-ankle inclination angle than the experienced group during the second half of the pre-swing phase. This suggests a different ability to handle the perturbation from the gesture leg in the two groups. Dancers used ankle plantarflexor moment, knee extensor moments, and hip flexor and abductor moments at the push leg (gesture leg) to initiate a turn (Zaferiou et al., 2017). The forces generated from the push leg (gesture leg) may further interfere with the dynamic balance of single-supporting leg in the frontal plane. Therefore, during pre-swing phase, dancers have to cope with the generated force by gesture leg and maintain balance in this transition phase.

### Retire Position

At the transition point between the pre-swing and mid-swing phases (i.e., the *retire position*), the novice dancers showed a significantly smaller absolute COM-ankle inclination_AP_ angle than the experienced group (Table 4). However, while the novice dancers had good postural stability, their *retire* performance was not as aesthetically pleasing as that of the experienced dancers. In general, the novice dancers showed a larger distance between the toe marker on the gesture leg and the medial knee marker on the supporting leg. In other words, the novice dancers were less accurate in their foot placement; perhaps as a result of a reduced proprioception of the lower extremity or a lower muscle effort by the hip abductors and external rotators (Bronner and Ojofeitimi, 2006). The increased hip muscle strength after ballet training (Bennell et al., 2001) suggests that the experienced dancers who have longer duration of ballet training may have better hip muscle strength to maintain their pelvis stability in *retire position* compared with the novice dancers. Furthermore, dancers with ballet training had a greater turnout angle than those who were not trained (Sutton-Traina et al., 2015). Thus, the experienced dancers may have a greater range of hip external rotation angle to maintain lateral-orientated thigh in *retire position*.

The novice dancers showed a smaller posterior COM-ankle inclination angle but greater medial inclination angle in the *retire position* than the experienced dancers (Table 4) due to a lower lateral thigh orientation of the gesture leg. In the ballet *retire* position, the thigh of the gesture leg should be as laterally oriented as possible in order to satisfy ballet aesthetics. A lower lateral orientation of the thigh leads to a greater mass transfer in the anterior direction, and thus results in a higher anterior and medial COM-ankle inclination angle in the novice dancers. Therefore, the present results suggest that novice dancers require reinforced stability training on single-leg support, with particular emphasis on appropriate thigh orientation and foot placement of the gesture leg in the *retire* position. In addition, a greater hip extensor and abductor moment of the support leg in *pirouettes* suggests that sufficient gluteal muscle strength is necessary for turning movements (Zaferiou et al., 2017).

### Turning With Single-Leg Support in Mid-swing Phase

**Figure 6:**
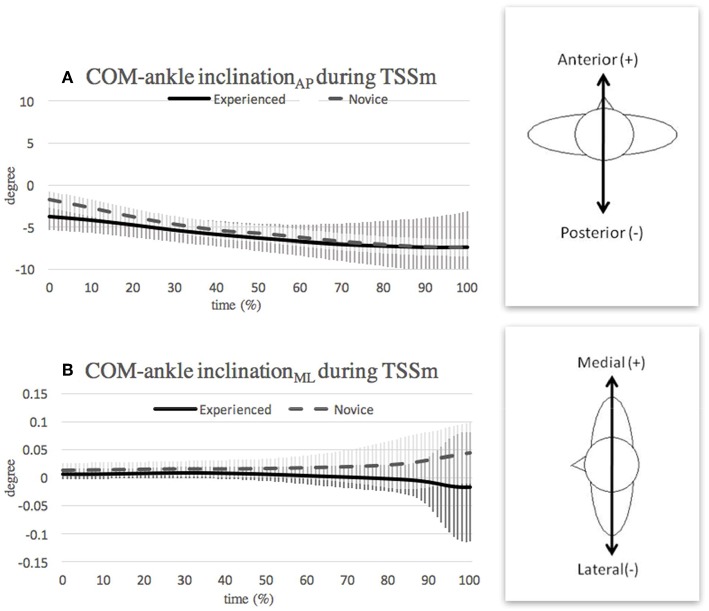
Mean and SD of COM-ankle inclination angle in **(A)** anterior-posterior direction and **(B)** medial-lateral direction during turning with single-leg support in mid-swing (TSSm) phase. Note that the curves show the average value obtained over the three highest-scoring trials of each individual in the respective group.

The placement of the gesture leg in the *retire position* affects the inclination angle in the first half of the subsequent mid-swing phase. Specifically, the lower lateral thigh orientation of the gesture leg in the novice dancers results in a greater anterior COM-ankle inclination angle (Figure 6A). During the second half of the mid-swing phase, the dancers position the foot of the gesture leg ready to perform the ending posture (i.e., ballet fifth *position*, with the gesture leg closely placed behind the supporting leg). Thus, for both groups, the COM-ankle inclination angle moves toward the posterior direction. As shown in Figure 6B, the experienced dancers showed both a smaller range and a smaller absolute value of the COM-ankle inclination_ML_ angle in the medial-lateral direction than the novice dancers almost throughout the entire mid-swing phase. This finding suggests that experienced dancers have an improved ability to align the COM vertically with the ankle joint while stabilizing their whole body in preparation for landing. Also, dancers who had the ability to execute greater numbers of turns in a *pirouette* allow body segment adjustments throughout the turn, instead of maintaining the trunk as a rigid body (Lott and Laws, 2012). This is because the coordination and adjustments of the body segments are essential for a high-skilled turn (Imura and Yeadon, 2010) and may again present better postural stability in preparation for landing.

### Ending Phase

**Figure 7:**
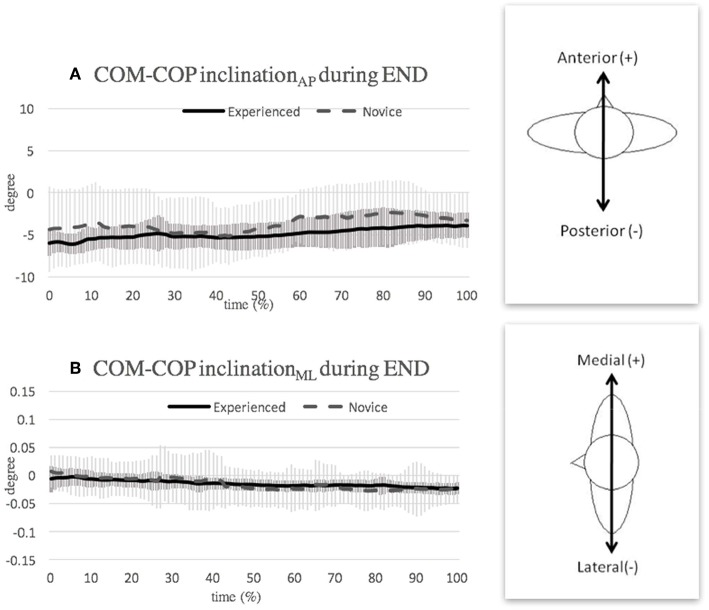
Mean and SD of COM-COP inclination angle in **(A)** anterior-posterior direction and **(B)** medial-lateral direction during ending (END) phase. Note that the curves show the average value obtained over the three highest-scoring trials of each individual in the respective group.

To satisfy ballet aesthetics, dancers are required to land gracefully at the end of the *pirouette*. However, the novice group exhibited a relatively large standard deviation of the COM-COP inclination angle in the ending phase (Figure 7); indicating a continuous adjustment of their body posture during landing. A greater postural adjustment was also observed in novice dancers during ellipse-drawing with an unloaded leg (Thullier and Moufti, 2004). A previous study shows that dancers used hip strategy to regain their balance as the difficulty of the task increases (Lott and Laws, 2012). The hip strategy is a way to maintain the COM over the base of support from a relatively large perturbation by using the hip as a fulcrum and bending the trunk. Thus, the novice dancers who had greater inclination angles in this study may take this hip strategy to maintain their balance in the ending phase. These findings, again, suggest that novice dancers are less skillful in performing ballet *pirouette*.

### Application

The present results show that in the preparatory phase of *pirouette en dehors*, novice dancers had slower responses than experienced dancers in modifying their posture in order to initiate the turning movement. Thus, it is suggested that novice dancers require specific training to ensure correct posture preparation prior to initiation of the turn. Furthermore, novice dancers require additional training to improve their speed of response to postural changes during the transition period from double-leg support to single-leg support, in order to improve their stability in this particular phase of the movement. Finally, in the *retire position*, a trend of lower lateral orientation of the thigh segment in novice dancers results in a greater anterior inclination angle. Consequently, specific training aimed at improving hip flexibility, hip extensors, abductors and external rotators strength, and foot placement is required.

### Limitations

In the present study, the experienced dancers had an age of 17.8 ± 3.4 years, whereas the novice dancers had an age of 12.0 ± 1.9 years. In practice, the age difference between the two groups is not easily avoided since ballet dancers generally begin at an early age; with the result that experienced dancers inevitably tend to be older than novices. In addition, some of the dancers adopted a leaping strategy trying to place their base of support under the COM in the single-leg support phase of the *pirouette*. While this study attempted to address this tendency by substituting the COP position with the midpoint position between the ankle joint center and the metatarsal marker during single-leg support, the effect of the leaping strategy on the present experimental findings cannot be precisely quantified. In the present study, the actions of the upper extremities were ignored as the initial training of ballet turns focused more on stability of the trunk and lower extremities. However, a previous study has shown that trail arm motion has contributed to generating angular momentum in *pirouette en dehors* (Kim et al., 2014). Another study looking at a ballet turn with relatively high technique, fouetté turn, suggested the contribution of upper extremities to the torso control (Imura and Yeadon, 2010). Future study should look into the influence of upper extremities on the ballet performance. Finally, the effects of age, gender and ethnicity on the moments of inertia of the different body segments of the dancers were not quantified in the present study.

## Conclusion

The movement strategies adopted by novice and experienced dancers in performing single-revolution ballet turns differ in terms of the COM-COP inclination angle and the COM-ankle inclination angle. Compared with experienced dancers, novice dancers exhibit overshooting errors and a greater COM-COP inclination angle during the preparatory phase. In addition, novice dancers maintain a lower lateral thigh orientation of the gesture leg (refers to less hip external rotation) in the ballet *retire position*, which is likely to result in a greater anterior inclination angle during the late pre-swing phase, *retire position*, and early mid-swing phase. Finally, novice dancers apply continuous postural adjustment during the ending phase compared with experienced dancers.

## Data Availability Statement

The datasets generated for this study are available on request to the corresponding author.

## Ethics Statement

The studies involving human participants were reviewed and approved by Institutional Review Board, National Cheng Kung University Hospital. Written informed consent to participate in this study was provided by the participants' legal guardian/next of kin.

## Author Contributions

C-WL involved in the design of study, data collection, data analysis, and manuscript writing. F-CS contributed to the design of study, data analysis, and manuscript writing. C-FL contributed to design of study, data collection, data analysis, and manuscript writing.

### Conflict of Interest

The authors declare that the research was conducted in the absence of any commercial or financial relationships that could be construed as a potential conflict of interest.
